# Characterizing responsive and refractory orthotopic mouse models of hepatocellular carcinoma in cancer immunotherapy

**DOI:** 10.1371/journal.pone.0219517

**Published:** 2019-07-10

**Authors:** Carina Hage, Sabine Hoves, Mailin Ashoff, Veronika Schandl, Stefan Hört, Natascha Rieder, Christian Heichinger, Marco Berrera, Carola H. Ries, Fabian Kiessling, Thomas Pöschinger

**Affiliations:** 1 Roche Innovation Center Munich, Roche Pharmaceutical Research and Early Development, Penzberg, Germany; 2 Institute for Experimental Molecular Imaging, University Clinic and Helmholtz Institute for Biomedical Engineering, RWTH Aachen University, Aachen, Germany; 3 Roche Innovation Center Basel, Roche Pharmaceutical Research and Early Development, Basel, Switzerland; Universidade de Sao Paulo, BRAZIL

## Abstract

Hepatocellular carcinoma (HCC) is one of the most common cancers worldwide and has a high mortality rate due to limited treatment options. Hence, the response of HCC to different cancer immunotherapies is being intensively investigated in clinical trials. Immune checkpoint blockers (ICB) show promising results, albeit for a minority of HCC patients. Mouse models are commonly used to evaluate new therapeutic agents or regimens. However, to make clinical translation more successful, better characterized preclinical models are required. We therefore extensively investigated two immune-competent orthotopic HCC mouse models, namely transplanted Hep-55.1c and transgenic iAST, with respect to morphological, immunological and genetic traits and evaluated both models’ responsiveness to immunotherapies. Hep-55.1c tumors were characterized by rich fibrous stroma, high mutational load and pronounced immune cell infiltrates, all of which are features of immune-responsive tumors. These characteristics were less distinct in iAST tumors, though these were highly vascularized. Cell depletion revealed that CD8^+^ T cells from iAST mice do not affect tumor growth and are tumor tolerant. This corresponds to the failure of single and combined ICB targeting PD-1 and CTLA-4. In contrast, combining anti-PD-1 and anti-CTLA-4 showed significant antitumor efficacy in the Hep-55.1c mouse model. Collectively, our data comprehensively characterize two immune-competent HCC mouse models representing ICB responsive and refractory characteristics. Our characterization confirms these models to be suitable for preclinical investigation of novel cancer immunotherapy approaches that aim to either deepen preexisting immune responses or generate de novo immunity against the tumor.

## Introduction

Hepatocellular carcinoma (HCC) is the most common liver cancer. Viral infections (hepatitis B, hepatitis C), alcohol consumption and non-alcoholic fatty liver diseases are the predominant risk factors [1]. HCC development follows a multistep pathological process: different genetic alterations contributing to liver injury and chronic inflammation are followed by dysplastic transformation of hepatocytes [1, 2]. Currently, patients diagnosed with HCC have a poor prognosis and usually receive palliative treatments [3, 4]. However, even the most powerful palliative drugs, such as the tyrosine kinase inhibitor (TKI) sorafenib, approved for advanced HCC in 2007, and the recently approved TKI lenvatinib only show limited efficacy [5, 6]. Immunotherapy is a promising alternative therapeutic strategy in HCC, and preclinical and clinical studies investigating immune checkpoint blockade (ICB) demonstrate better tumor shrinkage and improved overall survival [7, 8]. The PD-1 and CTLA-4 signaling pathways represent the most common targets of current immunotherapies. The PD-1 pathway suppresses T cell activation and proliferation, and the CTLA-4 pathway is involved in T cell priming in the lymph node [8]. A recent clinical trial of nivolumab targeting PD-1 in HCC patients revealed a response rate of 20% and a manageable safety profile emphasizing the importance of ICB for successful HCC treatment [8]. In 2017, the FDA approved nivolumab as a second-line therapy for HCC patients, who did not respond to first-line sorafenib treatment [9]. In November 2018, a second anti-PD-1 antibody, pembrolizumab, received approval for HCC patients, who have been previously treated with sorafenib [10]. Unfortunately, a significant portion of HCC still shows resistance to ICB [11]. Therefore, combination therapies are currently being explored in mice and humans and seem to be more potent than single agent administration [12].

Numerous mouse models have been developed to study human HCC and to investigate different therapeutic approaches [13]. Orthotopic models more closely resemble patient tumors than subcutaneous models because the tumors grow in their native environment. To induce orthotopic HCC, tumor cells can be injected directly into the liver [14, 15] or tumor fragments can be implanted intrahepatically [16, 17]. Mouse models can be distinguished in either xenograft models using immune-deficient mice [18] or syngeneic mouse models using immune-competent mouse strains such as C57BL/6. The latter are highly preferable for evaluating immunotherapies. In addition to syngeneic mouse models, humanized as well as genetically engineered models (GEM) can preclinically elucidate therapeutic immune responses against HCC [19]. Nevertheless, due to the multitude of etiological risk factors that underpin HCC recapitulate all descriptive features of the highly diverse HCC patient population.

In this study, we characterized the two different orthotopic HCC models, namely Hep-55.1c and iAST, [20] with respect to tumor growth, vascularization, morphology and immune infiltrate. Since mutational load is an important predictor of tumors’ susceptibility to immunotherapy we performed exome sequencing of the murine tumors to determine their mutational load and then compared them to human HCC. We also investigated the effect of two ICB targeting PD-1 and CTLA-4 in both models. The side by side evaluation highlights the most descriptive features and demonstrates that the established mouse models enable the investigation of novel HCC immunotherapies in tumors with either preexisting or lacking immunity.

## Results

### Orthotopic implantation of Hep-55.1c tumor cells and fragments leads to successful HCC tumor growth in the liver

**Fig 1:**
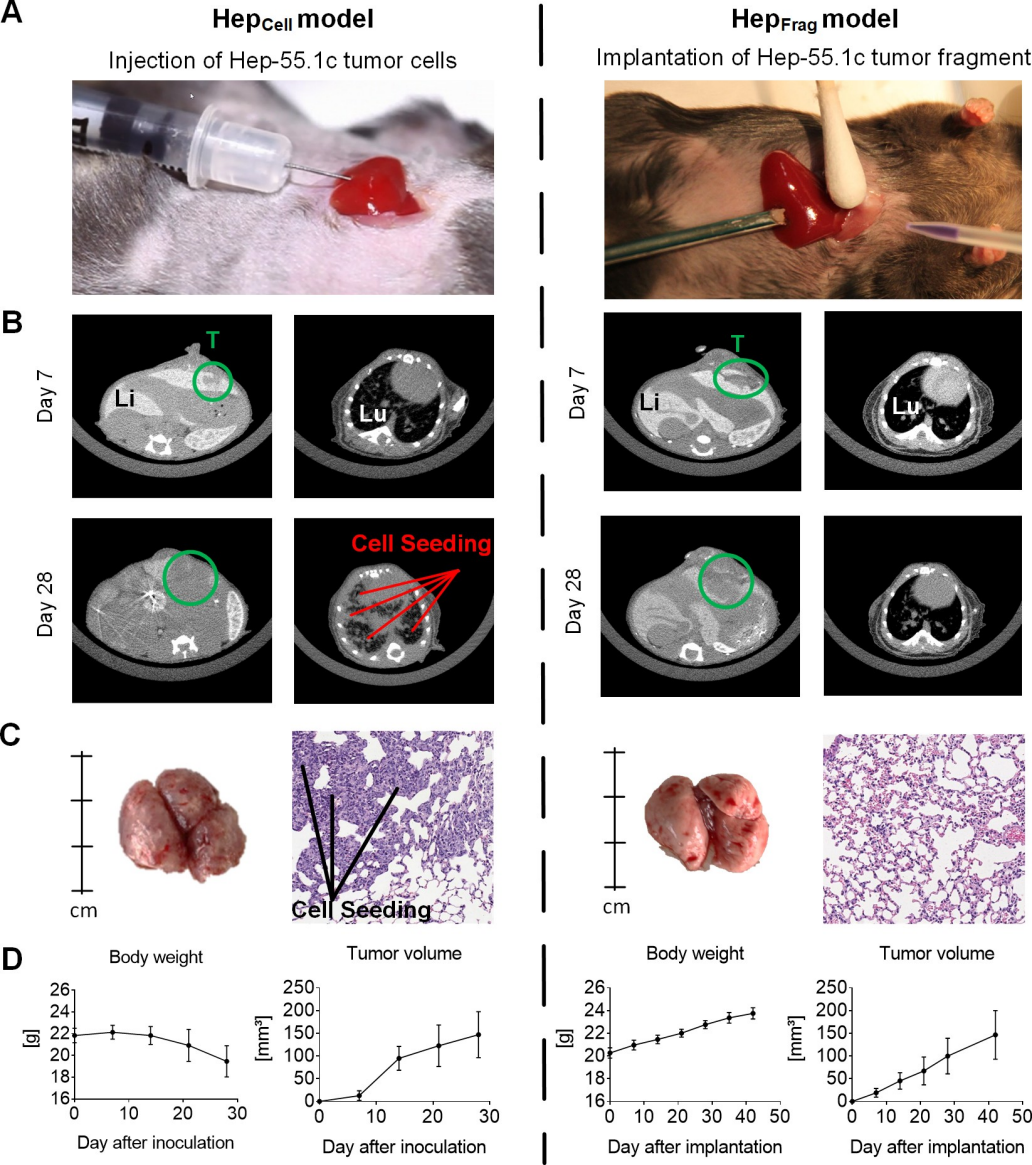
Establishing the orthotopic Hep-55.1c mouse model. A, Hep-55.1c tumor cells (left) or Hep-55.1c tumor fragments (right) were implanted into the left lateral liver lobe of C57BL/6 mice. B, Representative μCT images of tumors (T, green circle) in the liver (Li) and lung (Lu) on day 7 and day 28 after surgery. Lung images of Hep_Cell_ model on day 28 indicate cell seeding into the tissue (left). C, Representative images of explanted lungs on day 28 and HE-stained sections thereof illustrate the lung tumor burden in Hep_Cell_ mice (left). Lungs of Hep_Frag_ model explanted on day 35 after implantation did not show any tumor manifestation (right). D, Progressive body weight loss was observed in the Hep_Cell_ model within 28 days (left), whereas after Hep_Frag_ mice progressively gained weight within 42 days (right). Both models showed increasing tumor load over time (n = 5).

To generate an orthotopic HCC mouse model, Hep-55.1c tumor cells were inoculated into the left lateral liver lobe of immunocompetent C57BL/6 mice (Fig 1A, left). The growth of Hep-55.1c tumors in the liver was demonstrated by μCT imaging on days 7 and 28 after cell inoculation (Fig 1B, left). Of note, μCT imaging of the thorax also revealed tumor cell growth in the lung at day 28 after cell inoculation. The high tumor cell burden in the lung was confirmed by visual inspection after organ explantation and H&E staining of lung sections (Fig 1C, left). Body weight assessments revealed a loss of body weight within 28 days after surgery (Fig 1D, left), which might be attributed to cachexia at this high tumor load. The take-rate was 100% and tumor cell growth in the lung was detected in every mouse of the Hep-55.1c cell-based model.

Intrahepatic implantation of Hep-55.1c tumor fragments derived from Hep_Cell_ mice was performed using a trocar and tissue glue (Fig 1A, right). Liver tumor growth was detected by μCT at day 7 and day 28 after surgery (Fig 1B, right). No tumor growth in the lung tissue was observed by μCT. Lung explantation at day 35 followed by H&E staining of lung tissue sections confirmed the vital lung tissue architecture without any tumor cell burden in the Hep_Frag_ model (Fig 1C, right). Body weight analysis revealed a continuous increase over time (Fig 1D, right), which reflects tumor mass development in the liver without cachexia. Interestingly, tumors from Hep-55.1c fragments also grew more slowly than those from cell injection. All five Hep_Frag_ mice had visible tumors. These findings suggest that orthotopic fragment implantation precludes artificial tumor cell seeding in the lung and our study’s use of the significant early body weight loss as an endpoint criterion.

### Histological features of orthotopic HCC tumors

**Fig 2:**
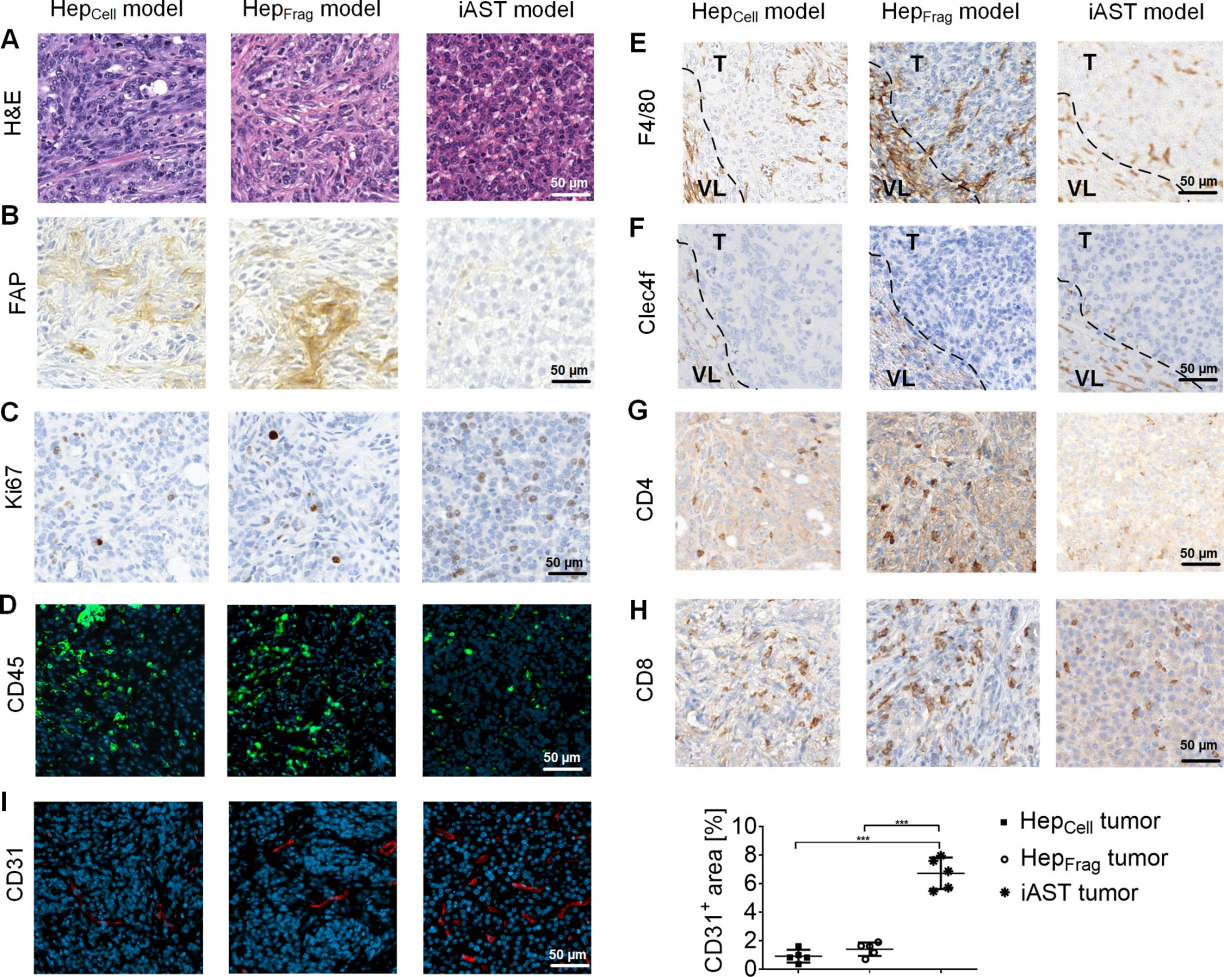
Histological analysis of orthotopic HCC mouse tumors. Hep_Cell_ tumors (day 21), Hep_Frag_ tumors (day 28) and iAST tumors (day 56) were stained for: A, morphology (H&E); B, tumor stroma (FAP); C, proliferation (Ki67); D, immune cells (CD45); E, macrophages (F4/80); F, Kupffer cells (Clec4f); G, CD4^+^ T cells (CD4); H, CD8^+^ T cells (CD8) and I, tumor vasculature (CD31). E,F, Dashed lines in the images of F4/80 and Clec4F stainings indicate the border between vital liver (VL) and tumor (T). I, CD31 signal quantification (n = 5) indicates significantly higher tumor vessel density in iAST compared to Hep tumors. In the scatter plot, each dot represents the average of 5 images per tumor. Differences between groups were tested for significance using the one-way ANOVA followed by Tukey multiple comparison test (***p<0.001).

Next, we evaluated Hep_Cell_ and Hep_Frag_ tumors histologically and compared them to transgenic iAST tumors. In this context, we let tumors from both models grow to a comparable size, at which sufficient tissue for our analyses was available but no major necrotic areas had developed. More specifically, Hep-55.1c tumors were explanted after reaching a size of approximately 100 mm³ (day 21 for Hep_Cell_ mice and day 28 for Hep_Frag_ mice). The iAST tumors were excised on day 56 upon virus injection as initial progression of this tumor is very slow compared to Hep tumors. H&E staining identified different morphologies among the tumor models (Fig 2A). FAP staining of the intratumoral extracellular matrix revealed that Hep_Cell_ tumors had a dense fibrotic network, which was even more pronounced in Hep_Frag_ tumors (Fig 2B). In contrast, iAST tumors were almost stroma-free. Subcutaneous Hep-55.1c tumors exhibit fibrotic structures similar to orthotopic Hep_Cell_ and Hep_Frag_ tumors (S1 Fig). Furthermore, iAST tumors were highly proliferative compared to Hep tumors as indicated by Ki67 stainings (Fig 2C). In line with this, iAST tumors were characterized by higher tumor cell density. Analyzing the total immune infiltrate by detecting CD45 showed more immune cells in Hep tumors compared to iAST tumors (Fig 2D). Hep tumors were rich in macrophages (F4/80^+^; Fig 2E), whereas Kupffer cells (Clec4F^+^) were located exclusively in the peritumoral vital liver tissue (Fig 2F). Also, the CD4^+^ (Fig 2G) and CD8^+^ T cell levels (Fig 2H) were slightly higher in Hep compared to iAST tumors. In addition, the tumors had different vascular architecture (Fig 2I). iAST tumors had significantly higher vessel density (CD31^+^) than Hep tumors. In addition, iAST tumors’ blood vessels were more homogeneous distributed. Taken together, these results show that Hep tumors and iAST tumors are different in morphology, stroma content, vascularization and immune cell composition. Serum analysis on day 56 upon virus injection reflects an increase of AST and ALT in iAST tumor-bearing mice compared to control iAST mice (S2 Fig).

### Hep tumors have higher immune infiltrate and mutational load than iAST tumors

**Fig 3:**
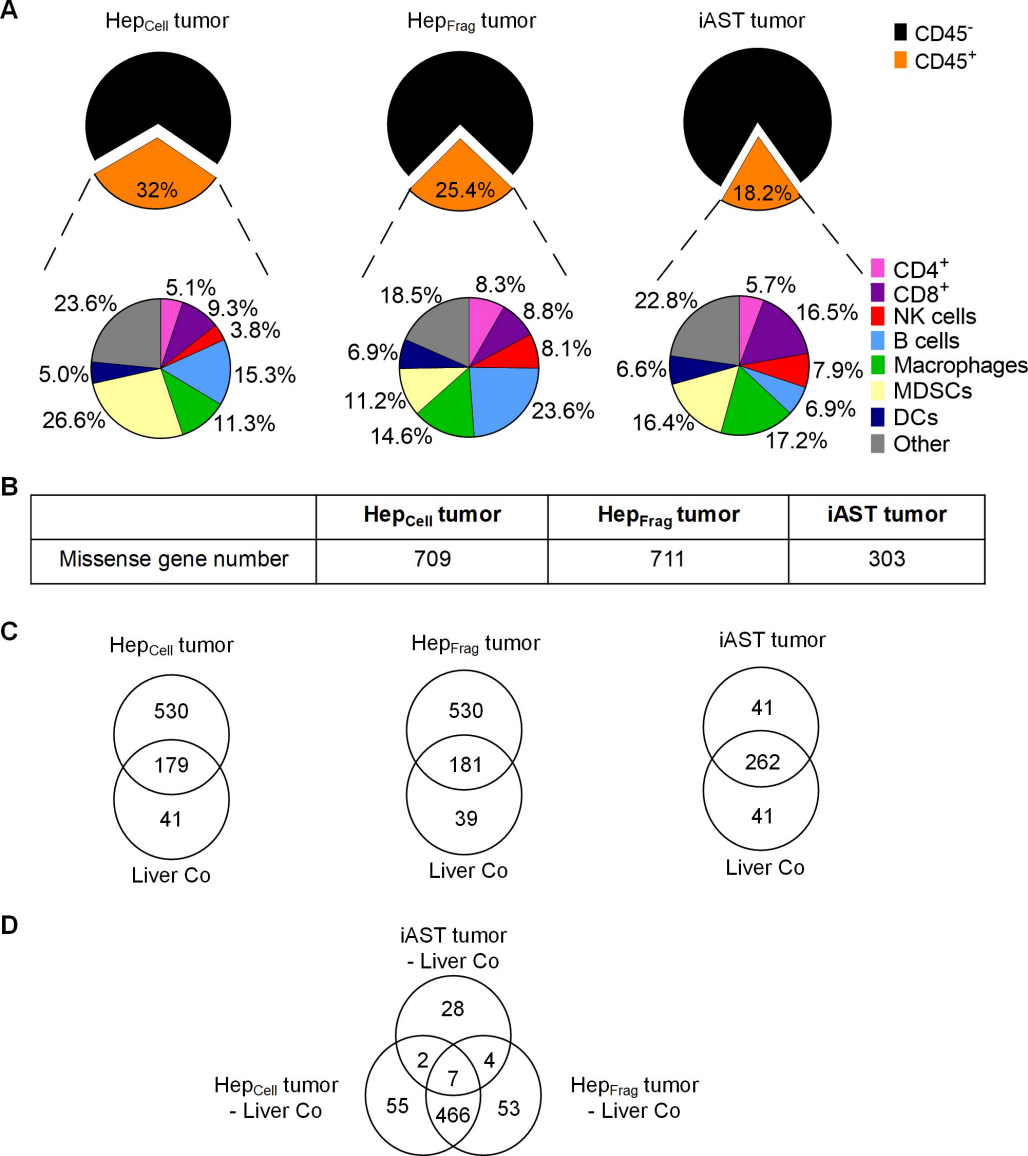
Model-specific immune cell infiltration and mutational load. A, Percentages of total immune cell infiltrate (CD45^+^) and subpopulations (CD4^+^, CD8^+^, NK cells, B cells, macrophages, MDSCs, DCs, other) in Hep_Cell_ tumors (day 21), Hep_Frag_ tumors (day 25) and iAST tumors (day 63). B, Exome sequencing of excised HCC tumors revealed considerably fewer lower number of missense genes in iAST (day 63) than in Hep_Cell_ (day 21) and Hep_Frag_ tumors (day 25). C, Venn diagrams show the mutated genes in each tumor with corresponding vital liver control (Liver Co). D, Venn diagram compares the mutated genes in the three tumors (Hep_Cell_, Hep_Frag_, iAST). Missense genes that were mutated in vital liver controls were subtracted from each tumor sample.

Next, we analyzed the murine HCC tumors’ immune infiltrate via flow cytometry (Fig 3A). iAST tumors had the lowest immune infiltrate, comprising 18.2% of total tumor cells whereas Hep_Cell_ and Hep_Frag_ tumors had 32.0% and 25.4%, respectively. Comparing the intratumoral immune cell subsets revealed that Hep_Cell_ tumors (26.6%) had significantly more myeloid derived suppressor cells (MDSCs) than Hep_Frag_ tumors (11.2%) and iAST tumors (16.4%). B cells were most frequent in Hep_Frag_ tumors (23.6%) compared to Hep_Cell_ tumors (15.3%) and iAST tumors (6.9%), respectively. iAST tumors had more CD8^+^ T cells (16.5%) than CD4^+^ T cells (5.7%), thereby confirming our histological analysis of T cells (Fig 2G and 2H).

We performed exome sequencing to gain further insight into the tumorigenicity and genetic characteristics of the mouse HCC tumors (Fig 3B–3D). Notably, iAST tumors had fewer missense genes (303) than Hep_Cell_ (709) and Hep_Frag_ tumors (711; Fig 3B). To dissect the tumor-specific mutations, we classified mutations appearing in vital liver tissue and in tumor tissue, respectively (Fig 3C). The overlap represents mutated genes detected in both, tumor and vital liver tissues. Of note, most of the mutated genes in iAST tumors were also present in the healthy liver sample (262 missense genes). In contrast, sequencing analysis of Hep tumors identified 530 genes mutated specifically in tumor samples indicating a high mutational load in Hep_Cell_ and Hep_Frag_ tumors.

We also categorized different tumor types’ mutations in a Venn diagram (Fig 3D). These data demonstrate that Hep_Cell_ and Hep_Frag_ tumors had a strong overlap in missense genes (466) but a very different profile than iAST tumors. Interestingly, only seven genes were mutated in all three tumor types. To identify frequent mutations in human HCC, we analyzed 373 human HCC samples from the cBio Cancer Genomics Portal; genes that were mutated in at least 8% of the samples are shown in Table 1.

**Table 1:** Mutated genes in human HCC. A total of 373 human HCC samples of the cBio cancer genomics portal were analyzed. The threshold for analysis was set for genes that were mutated in at least 8% of the samples.

Missense Gene	Gene Name	% in human HCC	Hep_Cell_ tumor	Hep_Frag_ tumor	iAST tumor
**TP53**	Transformation related protein 53	30.8	**X**	**X**	
**TTN**	Titin	27.1			
**CTNNB1**	Catenin beta 1	26			
**MUC16**	Mucin 16	14.5			
**ALB**	Albumin	11.5	**X**		
**APOB**	Apolipoprotein B	10.5			
**RYR2**	Ryanodine receptor 2, cardiac	10.5	**X**	**X**	
**PCLO**	Piccolo	9.4	**X**	**X**	**X**
**LRP1B**	Lipoprotein receptor-related protein 1B	8.8			
**CSMD3**	CUB and Sushi multiple domains 3	8.8	**X**	**X**	
**ARID1A**	AT-rich interactive domain-containing protein 1A	8.6			
**ABCA13**	ATP Binding Cassette Subfamily A Member 13	8.6	**X**	**X**	
**CACNA1E**	Calcium channel subunit alpha-1E	8.3			
**OBSCN**	Obscurin	8	**X**	**X**	
**RYR1**	Ryanodine receptor 1	8			

Next, we investigated whether the 15 missense genes in human HCC also occurred in mouse HCC tumors. Seven and six of the missense genes were discovered in Hep_Cell_ and Hep_Frag_ tumors, respectively. However, iAST tumors had only one mutation in line with the human HCC analysis (PCLO). The tumor suppressor gene TP53, which affects cell proliferation and apoptosis, is one of the most frequent mutations in cancer [21, 22] and appeared in 30.8% of the 373 human HCC samples. The TP53 mutation was found in both (Hep_Cell_ and Hep_Frag_ tumors). Interestingly, Kress et al. showed that the TP53 mutation was absent in the primary Hep-55.1c cell line [23], indicating that the gene mutated during cell passaging or development of mouse HCC tumors. The exome sequencing analysis revealed that Hep tumors contain more human HCC-specific missense genes than iAST tumors.

### Combined anti-PD-1 and anti-CTLA-4 treatment inhibits tumor growth in Hep-55.1c mouse model

**Fig 4:**
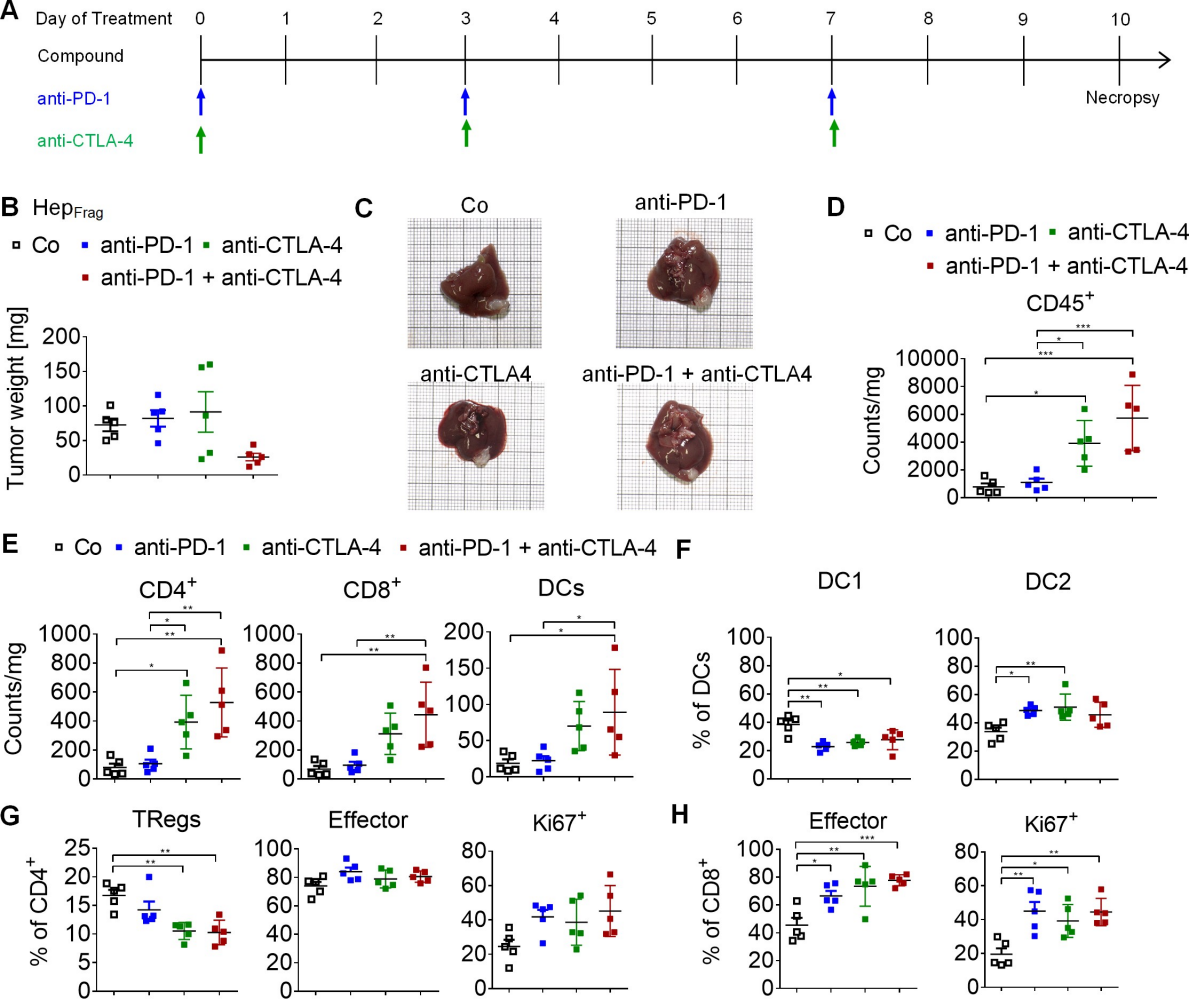
Anti-PD-1 and anti-CTLA-4 treatment of Hep_Frag_ tumors. A, Treatment schedule: anti-PD-1 (10 mg/kg, i.p.) and anti-CTLA-4 (5 mg/kg, i.p.) therapy started at day 25 after fragment implantation and was applied in 3 doses at days 0, 3 and 7. Necropsy of control (Co), anti-PD-1, anti-CTLA-4, and the anti-PD-1/anti-CTLA-4 combination groups (n = 5) was performed at day 10 after treatment initiation to assess tumor weight and immune cell infiltrates. B, Mice treated with combination of anti-PD-1 and anti-CTLA-4 show reduced tumor load compared to mice from the control and monotherapy groups. C, Representative images of explanted livers depicting the orthotopic tumor load of all groups. D, The total immune infiltrate in the tumors increased upon treatment with anti-CTLA-4 and the combination treatment with anti-PD-1. E, Similar increases were found for the total number of CD4^+^ T cells, CD8^+^ T cells and DCs upon combined treatment of anti-PD-1 and anti-CTLA-4. F, DC1 cells decrease upon treatment with anti-PD-1, anti-CTLA-4 and combination thereof, whereas an increase of DC2 cells was observed. G, The percentage of TRegs within CD4^+^ T cells decreases upon treatment with anti-CTLA-4 alone and in combination with anti-PD-1, whereas the content of CD4^+^ effector T cells and proliferating (Ki67^+^) CD4^+^ T cells did not significantly change upon treatment. H, Flow cytometry shows a significant increase in effector and proliferating (Ki67^+^) CD8^+^ T cells after anti-PD-1, anti-CTLA-4 and combined treatment. D-H, Differences among groups were tested for significance using the one-way ANOVA followed by Tukey multiple comparison test (*p<0.05, **p<0.01, ***p<0.001).

Although immunotherapy has been described as a promising treatment strategy for HCC [24, 25], in a large proportion of patients ICB shows little to no efficacy. In order to evaluate the response to ICB, we investigated the therapeutic effect of anti-PD-1, anti-CTLA-4 and the combination thereof in Hep_Frag_ mice (Fig 4A). After ten days, mice treated with a combination of anti-PD-1 and anti-CTLA-4 antibodies, showed reduced tumor load compared to mice treated with vehicle or respective monotherapy (Fig 4B and 4C). Analyzing the tumor immune infiltrate at treatment day 10 revealed a significant increase in CD45^+^ cells after both anti-CTLA-4 monotherapy and combination therapy with anti-PD-1 antibody. Furthermore, combined treatment with anti-PD-1 and anti-CTLA-4 antibodies produced higher CD4^+^ T cell, CD8^+^ T cell and DC infiltration into the tumor (Fig 4E). To investigate whether immune checkpoint inhibition also affects the DC subsets, we evaluated the CD11b^+^ DC1 population as well as the migratory CD103^+^ DC2 subtype (Fig 4F). Data showed a reduction of DC1 upon treatment and an increase in DC2, the latter are the pivotal DC population performing antigen cross presentation [26]. In addition, flow cytometry data indicated a reduced regulatory T cell population (TRegs) after anti-CTLA-4 monotherapy and combined treatment with anti-PD-1 (Fig 4G). CD4^+^ T cells were further characterized as effector T cells (~80% = CD44^+^/CD62L^-^) and proliferating CD4^+^ T cells tended to increase after both mono- and combination therapy. Analyzing the CD8^+^ T cell fraction revealed significant increase of effector cells and proliferating cells after treatment with anti-PD-1, anti-CTLA-4 and a combination thereof (Fig 4H). Collectively, these results indicate that combined anti-PD-1 and anti-CTLA-4 therapy produces a superior, more effective anti-tumor immune response compared to antibody monotherapy in Hep_Frag_ mice.

### Anti-PD-1 and anti-CTLA-4 treatments show no efficacy in iAST mouse model

**Fig 5:**
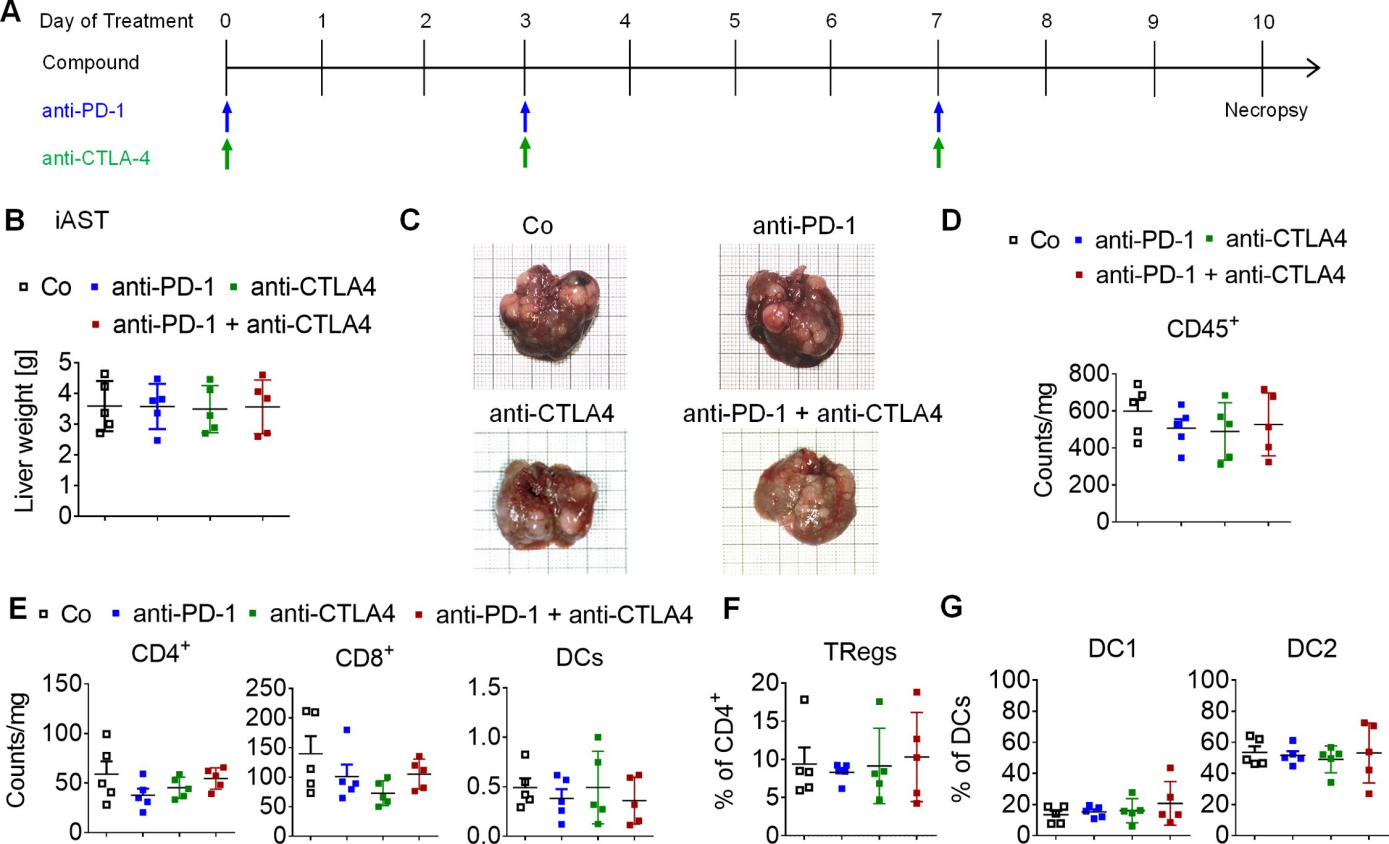
Anti-PD-1 and anti-CTLA-4 treatment of iAST tumors. A, Treatment schedule: anti-PD-1 (10 mg/kg, i.p.) and anti-CTLA-4 (5 mg/kg, i.p.) therapy started at day 53 after virus injection and was applied in three doses at days 0, 3, and 7. Necropsy of vehicle (Co), anti-PD-1, anti-CTLA-4 and combination group (n = 5) was performed at day 10 after treatment initiation. B, Treatment of mice with vehicle, anti-PD-1, anti-CTLA-4 or combination thereof did not alter tumor load. C, Representative images of explanted livers illustrate multinodular tumors after treatment with vehicle, anti-PD-1, anti-CTLA-4 or combination thereof. D, Analysis of the total immune infiltrate and subsets in the tumor nodules did not show any significant differences among the groups. E, The total number of CD4^+^ T cells, CD8^+^ T cells and DCs did not change after treatment with anti-PD-1, anti-CTLA-4 or combination thereof (n = 5). F, Percentage of TRegs within the CD4^+^ T cell population did not change upon treatment. G, The distribution of DC subsets was not affected by treatment. D-G, Differences among groups were tested for significance using the one-way ANOVA followed by Tukey multiple comparison test.

In order to investigate the efficacy of ICB in the multinodular HCC tumors of the iAST model, mice were treated with anti-PD-1 and anti-CTLA-4 monotherapy and in a combined setting (Fig 5A). Treatment was initiated on day 53 after virus injection when multiple tumors have been established within the liver (S3 Fig). Explanting livers, including multinodular tumors, revealed no differences in tumor load at day 10 of treatment (Fig 5B and 5C). The number of intratumoral immune cells determined via flow cytometry did not change upon treatment (Fig 5D). Further analysis of immune cell subsets revealed similar amounts of CD4^+^ T cells, CD8^+^ T cells and DC in all treatment groups (Fig 5E). Moreover, the TReg cell population (Fig 5F) and the distribution of DC subsets (Fig 5G) were not affected by treatment with anti-PD-1 and anti-CTLA-4 mono- and combination therapies. These data indicate that iAST tumors did not respond to ICB using anti-PD-1 and anti-CTLA-4 antibodies. To investigate the impact of cytotoxic T cells on iAST tumor growth, CD8^+^ T cells were depleted (S4 Fig). Measuring liver weight after explantation revealed no difference in multinodular tumor load between the control (IgG) and depletion groups (CD8^-^), indicating that cytotoxic T cells do not affect tumor development in iAST mice (S4B Fig). In the literature, the PD-L1 expression in the tumors is often described to be a predictive biomarker for effective anti-PD-1 therapies [27]: accordingly PD-L1 expression was higher in Hep-55.1c tumor sections than iAST tumor sections (S5 Fig).

## Discussion

Multiple HCC mouse models are used preclinically to assess response to new therapeutic compounds and to identify prognostic factors [28, 29]. To our knowledge, however, there has been neither a detailed characterization of HCC mouse models’ specific features in comparison to human HCC nor an evaluation of their suitability for preclinically testing novel cancer immunotherapies. To address this gap in knowledge, we comprehensively characterized two orthotopic HCC models (Table 2) and investigated their responses to ICB. Female mice were used in this study due to their more social behavior compared to male mice.

**Table 2:** Characteristics of orthotopic HCC mouse models.

	Hep_Cell_ model	Hep_Frag_ model	iAST model
**Multinodular growth**	No	No	Yes
**Cell seeding in the lung**	Yes	No	No
**Body weight loss**	Yes	No	No
**Tumor vascularization**	Moderate	Moderate	High
**Tumor stroma intensity**	High	High	Low
**Tumor immune infiltrate**	High	High	Moderate
**Tumor mutational load**	High	High	Low
**Response to checkpoint blockade**	*not determined*	Yes	No

Following orthotopic injection of Hep-55.1c tumor cells, we observed a tumor take rate of 100% with a short tumor development latency. At the same time, cell inoculation into the liver lobe led to tumor cell dissemination into the lung. The formation of multiple lung tumors induced cachexia and severely reduced lifespan. The strong tumor dissemination and rapid growth makes this model rather artificial, and the short life span of animals limits the treatment window to assess drug responses. Furthermore, human HCC rarely form lung metastases [30]. Nonetheless, the Hep_Cell_ model can also be used as preclinical lung metastasis model. The implantation of Hep-55.1c tumor fragments into the liver of C57BL/6 mice also caused progressive primary tumor growth but circumvented the random tumor cell seeding in the lung tissue and thus was considered preferable. In our opinion, the Hep_Cell_ and Hep_Frag_ models correspond to the less frequent situation of solitary HCC in patients whereby the Hep_Frag_ model reflects HCC without any tumor cell seeding in the lung [31]. In contrast, the transgenic iAST model requires the application of the Cre recombinase to generate a multinodular HCC tumor growth in its natural environment. Compared to other GEM models, tumors of iAST mice grew rather quickly within 6–7 weeks after virus injection, and have a tumor take rate of 100%. The adenovirus application induces the development of hepatitis followed by damage to liver parenchyma [32]. Yet iAST tumors’ aggressive development distinguishes them from human HCC, which typically progress slowly [28].

Histologically, the different tumor types reflect different HCC specific traits. Most human HCC are characterized by a strong intratumoral vascularization [12, 33]. This feature was rather pronounced in iAST tumors in contrast to the lower vessel density in Hep_Cell_ and Hep_Frag_ tumors. Another typical feature of human HCC is that they frequently develop as a result of chronic liver damage and contain a high number of stroma cells [34]. Analysis of the extracellular matrix of mouse HCC tumors revealed that Hep-55.1c tumors have distinct stroma regions composed of fibroblasts and extracellular matrix (ECM), whereas iAST tumors lack e.g. expression of FAP in the tumor microenvironment. As stroma regions are also present in subcutaneous Hep-55.1c tumors the development of the desmopastic framework might not be caused by surgical intervention. Tumor desmoplasia can affect immune cell infiltration and reasons for its development remain unclear [35]. On the one hand, an early immune reaction may lead to scaring and thus a stroma-rich tumor. Alternatively, fibroblasts may be actively recruited by the tumor cells leading to enhanced ECM production to protect tumors from immune cell infiltration. Whichever hypothesis is correct, the lack of fibrous stroma in iAST tumors is a further indicator that this model is missing an immune reaction. In line with this, a richer immune infiltrate was found in Hep_Frag_ tumors and Hep_Cell_ tumors compared to iAST tumors. Published data indicate a correlation between intratumoral T and B cells correlate with a positive clinical outcome of HCC patients [36]. Strikingly, iAST tumors were found to be highly infiltrated by CD8^+^ T cells, the key effector cells of most cancer immunotherapy approaches [37]. However, the cell depletion experiment provided evidence that CD8^+^ T cells in iAST mice did not affect tumor development. In inducible mouse models based on the Cre/loxP system the transgenic oncogene (SV40 T antigen) is presented as self antigen [20]. In this context, Willimsky et al. have previously demonstrated that an incomplete function of the stop cassette leads to T cell tolerance towards the antigen before the onset of tumor development [38]. Mice with tolerant T cells did not induce functional cytotoxic T lymphocytes and were thus unable to reject the tumor. This characteristic of CD8^+^ T cells corresponds to cancer patients who are not responsive to cancer immunotherapy due to the presence of dysfunctional T cells in the tumor microenvironment [39, 40]. In a variety of HCC patients, chronic inflammation and impaired co-stimulatory signals lead to T cell anergy or exhaustion [41].

Differences in the immune cell infiltrate can also be attributed to the genetic characteristics of HCC and are, therefore, major factors in the response to immunotherapy [42]. HCC tumors consist of a heterogenic group of cells that can exhibit numerous mutations [28]. A typical genetic alteration detected in human HCC patients is found in the TP53 gene [12, 43]. Here, sequencing the murine tumors revealed a variety of genetic alterations in Hep_Cell_ and Hep_Frag_ tumors including the common TP53 mutation. In contrast, iAST tumors show a low mutational burden, so that this model corresponds to human tumors with low numbers of genes carrying missense mutations and weak sensitivity to ICB [44].

The preclinical evaluation of anti-PD-1 and anti-CTLA-4 mono- and combination therapies revealed different responses of Hep_Frag_ and iAST tumors. Combining both antibodies, anti-PD-1 and anti-CTLA-4, led to tumor shrinkage in Hep_Frag_ tumors, whereas the monotherapy had no beneficial effect on tumor regression. These data accord with previous studies indicating that anti-PD-1 monotherapy shows no therapeutic efficacy in Hep-55.1c tumor-bearing mice [45]. The superior outcome of dual checkpoint blockade has already been confirmed in clinical trials on different cancer types. In advanced melanoma the combination of nivolumab (anti-PD-1) and ipilimumab (anti-CTLA-4) resulted in a response rate of 53% and had a manageable safety profile similar to respective single antibody administrations [46, 47]. This synergy and consequent enhanced anti-tumor responses are currently under investigation in a prospective Phase I/II study for advanced HCC (NCT01658878) [4]. The response to combined ICB targeting PD-1 and CTLA-4 may be explained by the mutational burden of Hep_Frag_ tumors and the pre-existing immunity in this model. The initial immunogenic character of transplanted tumors was described recently [48].

Given the aforementioned fundamental differences in the composition of the tumor microenvironment of both HCC mouse models, the failure of single and combined ICB targeting PD-1 and CTLA-4 in iAST mice is not surprising and can be explained predominantly by the low number of missense genes and previously demonstrated T cell tolerance within the tumors. In addition, our data revealed very low PD-L1 expression in iAST tumors compared to Hep_Frag_ tumors, which indicates that T cell inhibition is not dominantly mediated by PD-1/PD-L1 interaction. Different therapeutic strategies to overcome CD8^+^ T cell tolerance and induce antitumor immunity are currently under investigation [37]. Cancer vaccines, CAR T cells and adoptive transfer of immune effector cells are promising approaches to provide tumor specific immune cells and mediate tumor regression [49]. Another effective option to treat HCC in immune-tolerant models is the TKI sorafenib, which has been shown to inhibit tumor growth and prolong survival of iAST mice [32, 50].

In summary, we here provide a comprehensive characterization of the orthotopic Hep-55.1c and of the iAST mouse models with regard to their suitability for preclinically testing new HCC treatment strategies, particularly cancer immunotherapies. We identified the Hep-55.1c model as responsive to combined ICB targeting PD-1 and CTLA-4. In contrast, the iAST model was shown to mirror ICB refractory characteristics, which renders the iAST model useful for testing cancer immunotherapy and combination treatments beyond ICB in order to overcome the HCC induced immune tolerance. In conclusion, increased knowledge of the fundamental characteristics of HCC mouse models is essential to correctly understand the mechanisms of cancer immunotherapies and thus, guide the treatment of HCC patients based on scientific rationale.

## Materials and methods

### Mice and cell lines

Female mice (C57BL/6 wildtype or iAST transgenic mice) were obtained from Charles River Laboratories (Sulzfeld, Germany). The animal facility has been accredited by the Association for Assessment and Accreditation of Laboratory Animal Care (AAALAC). All animal studies were performed in accordance with the Federation for Laboratory Animal Science Associations (FELASA). Mice were euthanized by cervical dislocation. A body weight loss of 20% was defined as an endpoint criterion. The animal studies were approved by and done under license from the Government of Upper Bavaria (Regierung von Oberbayern; Approval number: ROB-55.2-2532.Vet_03-15-89).

The HCC tumor cell line Hep-55.1c (Hep) was obtained from Cell Line Services (Eppelheim, Germany) and cultured with DMEM high glucose medium (PAN Biotech) supplemented with fetal calf serum (10%, Gibco) and L-glutamine (5%, PAN Biotech). Authenticity control of the Hep cell line was performed by DSMZ (Leibniz Institute Braunschweig, Germany). Polymerase chain reaction (PCR) was performed by Charles River Laboratories (Wilmington, MA, USA) to confirm the absence of mycoplasma.

### Establishment of Hep-55.1c mouse model

In order to generate Hep-55.1c tumor-bearing mice, either, Hep-55.1c tumor cells or tumor fragments were implanted, respectively, into C57BL/6 wildtype mice. For the Hep-55.1c cell-based model (Hep_Cell_ model), 8–9 week old mice weighing 21–23 g were used. The mouse abdomen was shaved and animals were treated subcutaneously with the analgesics rimadyl (5 mg/kg; Zoetis, Berlin, Germany) and metamizol (100 mg/kg; WdT, Garbsen, Germany) 30 min prior to surgery as well as 24 and 48 hours after surgery. For surgical inoculation, animals were anesthetized with 2% isoflurane at 2 L/min oxygen and mouse eyes were covered with dexpanthenol eye ointment (Bepanthen, Roche, Grenzach-Wyhlen. Germany) to avoid dehydration of the cornea. A 5 mm subcostal incision was made into skin and peritoneum to uncover the liver, and a total of 5x10^5^ Hep-55.1c cells in 20 μL matrigel (Corning, Bedford, MA, USA) were injected into the left lateral liver lobe using an insulin syringe (31 G, BD, Franklin Lakes, NJ, USA). The injection site was covered with tabotamp (Ethicon, Norderstedt, Germany) and dabbed with a cotton swab of betaisadona (Mundipharma, Limburg, Germany). The peritoneum was closed with absorbable suture material (Prolene, Ethicon, Norderstedt, Germany) and skin was clamped.

For the Hep-55.1c fragment-based mouse model (Hep_Frag_ model), 6–8 week old mice weighing 19–22 g were used. Tumor fragments for implantation were generated from orthotopic Hep-55.1c tumors that were induced by cell injection (donor animals). Tumors were extracted 20 days after inoculation and cut into tissue fragments of 1.8 mm diameter under sterile conditions. In each recipient mouse, one Hep-55.1c tumor fragment was implanted into the left lateral liver lobe by using a sterile trocar and the fragment was fixed with one drop of bio-degradable tissue glue (Histoacryl, B.Braun, Melsungen, Germany). Surgery (anesthesia, analgesia, wound sealing) was performed as described above for the Hep_Cell_ model.

### Tumor growth induction in orthotopic iAST mouse model

For the orthotopic iAST mouse model, inducible AST (iAST) mice were used (as described in [20]). The iAST mice express the SV40 large T antigen with a hepatocyte-specific albumin promoter. Conditional expression is regulated by a loxP flanked stop cassette. Tumor growth was induced by intravenous (i.v.) injection of 5x10^8^ infectious units (IU) of adenovirus (Ad.Cre) expressing Cre recombinase (Vector BioLabs, Malvern, PA, USA) into 6–8 week old iAST mice.

### Micro-computed tomography (μCT)

Orthotopic tumor growth was determined by *in vivo* μCT imaging. Image acquisition was done with a μCT device (TomoScope Synergy Twin, CT Imaging GmbH, Erlangen, Germany). Liver contrast was enhanced by injecting 100 μL of ExiTron nano 6000 (Viscover, Miltenyi Biotec, Bergisch Gladbach, Germany) at least 4 hours before first image acquisition. μCT scans were performed using a high resolution protocol (parameters: 1440 projections, tube voltage = 50 kV, tube current = 0.8 mA scan time = 180 s). Prior to scanning, mice were anesthetized with 2% isoflurane at 2 L/min oxygen. Volumetric images were reconstructed using a cone-beam Feldkamp algorithm and images were visualized using the software OsiriX (Pixmeo, Bernex, Switzerland).

### Immunohistochemistry (IHC)

Tumors were harvested, fixed in formalin, dehydrated in ethanol and xylol series and then embedded in paraffin. Formalin-fixed, paraffin-embedded tissue (FFPET) sections (2.5 μm thick) were stained with hematoxylin and eosin and immunohistochemistry was performed using either a VENTANA Discovery XT (NEXES software v10.6) or a BenchMark ULTRA (VSS software v12.3) automated slide stainer. The following anti-murine primary antibodies were used: Ki67 (rabbit monoclonal, clone 30–9, Ventana Medical Systems), F4/80 (rat monoclonal, clone BM8, Acris Antibodies GmbH), Clec4f (goat polyclonal, R&D Systems), CD4 (rabbit monoclonal, clone #1, Sino Biological Inc), CD8α (rat monoclonal, clone GHH8, Dianova), PD-L1 (rabbit monoclonal, clone E1L3N, Cell Signaling). Each primary antibody was detected with a corresponding anti-species secondary HRP multimer (anti-rabbit/rat/goat OmniMap-HRP, Ventana Medical Systems). Signal detection was performed with DAB chromogen using either ChromoMap DAB or OptiView DAB detection kits (Ventana Medical Systems), and slides were counterstained with hematoxylin. To evaluate FAP, cryostat sections (8 μm thick) of fresh frozen tumor tissues were generated. Sections were stained with anti-FAP (rabbit monoclonal, clone 28H1, Roche Glycart AG) using a VENTANA Discovery XT automated stainer (using anti-rabbit UltraMap-HRP and ChromoMap DAB detection kit). Stained slides were scanned using an iScan HT scanner (Ventana Medical Systems).

### Immunofluorescence (IF)

FFPET sampes were cut into 1.5 μm sections. Samples were deparaffinized in a descending xylene and ethanol series and rehydrated in deionized water for 30 s. Subsequently, antigen retrieval and protein blocking (Dako) were conducted. Anti-mouse CD31 (clone SZ31, Dianova) and anti-mouse CD45 antibodies (clone 30-F11, eBioscience) were applied as primary antibodies. The primary antibody incubation (1 hour) was followed by incubation with Alexa647-labeled secondary antibody (Thermo Fisher) for 30 min in dark. Subsequently, the sections were covered with a DAPI-containing mounting medium (Fluoro-Gel II, Electron Microscopy Sciences, Hatfield, PA, USA). Fluorescence measurements of slides were performed using a slide scanner Pannoramic 250 Flash (3D Histech, Budapest, Hungary). Image visualization was done with the software Pannoramic Viewer (3D Histech, Budapest, Hungary).

### Flow cytometry

Single cell suspensions of explanted tumors were generated and processed according to the following protocol. For the multinodular iAST model, three nodules were pooled per mouse. Tumors were mechanically processed in a petri dish using scalpel and forceps. Samples were digested at 37°C for 30 min with the enzymes DNAse I (0.01%; Roche) and collagenase IV (1 mg/mL; Sigma). Red blood cells were lysed using lysing buffer (BD Biosciences) for 5 minutes at room temperature (RT). Cell numbers in the single cell suspensions were determined with the ViCELL analyzer (Beckman Coulter, High Wycombe, UK) and 1x10^6^ cells per sample were transferred to 96-well plates. Fc receptors were blocked with rat anti-mouse FcγIII/II receptor (CD16/CD32) blocking antibody (4 μg/mL, clone 2.4G2, BD Biosciences) for 5 min on ice. The following antibodies (clones) were used for cell staining: CD45 (30-F11), CD11b (M1/70), CD3 (17A2), CD4 (RM4-5), CD8α (53–6.7), F4/80 (BM8), NK1.1 (PK136), Ly6G (1A8), Ly6C (AL-21), MHC class II I-A/I-E (M5/114.15.2), CD19 (6D5), CD11c (N418), CD24 (M1/69), CD103 (2E7), CD25 (PC61), CD279 (29F.1A12), CD44 (IM7), CD62L (MEL14) as well as matching isotype controls (all from BioLegend or BD Biosciences). Intracellular staining with anti-FoxP3 (MF14) or anti-Ki67 (16A8) antibody was performed according to the manufacturer’s instructions from the intracellular fixation and permeabilization kit (eBioscience). DAPI (Roche) or fixable Zombie UV dye (BioLegend) was used to determine cell viability. All samples were measured using a LSRFortessa flow cytometer (BD Biosciences), and data were analyzed with FlowJo software (version 10, Treestar). Cells were identified using the following combination of cell markers after gating on single cells and discriminating between live and dead cells: total immune infiltrate as CD45^+^; CD4^+^ T cells as CD45^+^CD11b^−^CD3^+^CD4^+^; CD8^+^ T cells as CD45^+^CD11b^−^CD3^+^CD8a^+^; Regulatory Cells (TRegs) as CD45^+^CD11b^−^CD3^+^CD4^+^FoxP3^+^CD25^+^; myeloid derived suppressor cells (MDSC) as CD45^+^CD11b^+^ F4/80^-^Ly6C^high^ Ly6G^-^ and CD45^+^CD11b^+^F4/80^-^Ly6C^low^Ly6G^+^; Macrophages as CD45^+^CD11b^+^F4/80^+^; NK cells as CD45^+^NK1.1^+^; B cells as CD45^+^CD19^+^; dendritic cells (DC) as CD45^+^Ly6C^-^Ly6G^-^MHC class II^+^F4/80^low^CD24^+^; DC1 as CD45^+^Ly6C^-^Ly6G^-^MHC classII^+^F4/80^low^CD24^+^CD11b^+^; DC2 as CD45^+^Ly6C^-^Ly6G^-^MHCclassII^+^F4/80^low^CD24^+^CD103^+^; CD4^+^ effector T cells as CD45^+^CD11b^−^CD3^+^CD4^+^CD44^+^CD62L^-^; CD8^+^ effector T cells as CD45^+^CD11b^−^CD3^+^CD8a^+^ CD44^+^CD62L^-^.

### Exome sequencing

The AllPrep DNA/RNA Mini Kit (Qiagen) was used to co-extract DNA and RNA from mouse liver and tumor sections. Up to 30 mg of tissue was homogenized in 600 μL of RNA lysis buffer (including beta-mercaptoethanol) using the Qiagen tissue lyser for 2 x 2 min at 20 Hz. The lysate was centrifuged for 3 min at 14x10^3^ rpm at RT. The standard Qiagen AllPrep DNA/RNA co-extraction protocol was then used for genomic DNA extraction. DNA quality was assessed on the Agilent Tapestation (DIN 7.2–8.7). For whole exome sequencing, the NimbleGen SeqCap EZ Library SR User Guide V5.1 was followed using the SeqCap EZ Developer Library (110624_MM9_exome_L2R_D02_EZ_HX1) to enrich for mouse exome sequences. Three to four samples were multiplexed before hybridization to the developer library. The exome sequencing library yield and quality were assessed using the DNA1000 Bioanalyzer assay. Exome enrichment for all exome libraries met QC requirements as assessed by the qPCR assays suggested in the SeqCap protocol. Libraries were sequenced on the Illumina HiSeq4000 system, paired-end 2 x 151 cycles. Between 67.5 and 78 million paired-end reads were mapped to the mouse genome for each sample.

### Bioinformatics analysis

Output data from exome sequencing were mapped on the *Mus musculus* genome draft GRCm38/mm10 using the program BWA (version 0.7.16a-r1181) resulting in ~90% mapping rate for each sample. Read duplicates were removed using the program PICARD (version 1.39). Base realignment and calibration as well as variant calling were done using the program GATK (version 3.7), while variant annotation was performed via the program snpEff (version 4.3) with the GRCm38.86 annotation ignoring known variants in mouse strain C57BL/6NJ as from dbSNP Build 142.

To compare the murine sequencing results with human cancer genomics data sets, 373 HCC samples obtained from the cBio Cancer Genomics Portal (http://cbioportal.org) were analyzed [51–53]. Missense genes, which were detected in at least 8% of human HCC samples, were determined and compared to mutations in murine samples.

### *In vivo* treatment

Mice were treated with anti-PD-1 (clone RMP1-14, 10 mg/kg), anti-CTLA-4 (clone 9D9, 5 mg/kg) or both. The rat IgG2a antibody (clone 2A3) served as isotype control. Treatment began at day 30 after fragment implantation for Hep-55.1c mice and at day 53 after virus injection for iAST mice, respectively. All compounds were administered intraperitoneally (i.p.) at indicated time points. All antibodies were obtained from BioXCell.

### *In vivo* CD8^+^ T cell depletion

CD8^+^ T cell depletion experiments were performed on iAST mice 49 days after virus injection. Mice were treated i.p. at days 49, 51, 53, 56, 59 and 62 with 4 mg/kg anti-CD8α antibody (clone 53–6.7, BioXCell). Rat IgG2a antibody (clone 2A3, BioXCell) was injected in parallel and served as isotype control. Cell depletion was confirmed by flow cytometry analysis of iAST tumors using the anti-CD8α antibody (clone 5H10, BioLegend).

### Statistical analysis

Two group comparisons were performed using the Student’s t test. For experiments with more than two groups, statistical analysis was performed by one-way analysis of variance (ANOVA) followed by Tukey multiple comparison test. Statistical significance was indicated as follows: * = 0.01 ≤ p < 0.05; ** = 0.001 ≤ p < 0.01; *** = p < 0.001. For statistics and graphs the software GraphPad Prism (version 6, GraphPad Software Inc., San Diego, CA, USA) was used.

## Supporting information

S1 Methods (DOCX)

S1 Fig Stroma content in subcutaneous Hep-55.1c tumors. H&E staining of subcutaneous Hep-55.1c tumor of day 21 after cell inoculation.
(TIF)

S2 Fig AST and ALT levels in serum of iAST mice. Analysis of liver enzymes AST and ALT in serum of iAST control mice and tumor-bearing mice of day 56 after virus injection.
(TIF)

S3 Fig Tumor growth in iAST model. Representative μCT images of an iAST mouse on day 53 and day 58 after virus injection.
(TIF)

S4 Fig CD8^+^ T cell depletion does not affect iAST tumor growth. A, Treatment schedule illustrating the days of CD8^+^ T cell depletion (Day 0, 2, 4, 7, 10, 13) using a CD8α T cell depletion antibody (4 mg/kg, i.p.). Necropsy of mice was performed at day 14 after treatment initiation. B, Assessment of the weight of explanted livers including multinodular HCC tumors did not show a change in tumor load after depletion compared to control IgG (n = 6). C, Flow cytometry analysis confirms the successful depletion of CD8^+^ T cells in iAST tumors. B-C, Comparisons between groups were performed by Student’s t-test (***p<0.001).
(TIF)

S5 Fig PD-L1 expression in Hep_Frag_ and iAST tumors. Representative tumor sections of Hep_Frag_ (day 28) and iAST tumors (day 56) were stained for PD-L1. A higher PD-L1 expression was observed in Hep_Frag_ tumors as compared to iAST tumors.
(TIF)
