# Supplementary material for: Characterizing responsive and refractory orthotopic mouse models of hepatocellular carcinoma in cancer immunotherapy
Source: PLoS One. 2019 Jul 10;14(7):e0219517. doi: 10.1371/journal.pone.0219517 (PMC6619768; doi:10.1371/journal.pone.0219517)
Supplement: S1 Methods — (DOCX) [file pone.0219517.s001.docx]

# Supporting Materials and methods

## **Establishment of subcutaneous Hep-55.1c tumors**

In order to establish subcutaneous Hep-55.1c tumors in C57BL/6 mice, Hep-55.1c cells (5 x 10^6^ cells in 100 µL PBS) were injected subcutaneously into the right flank.

## **Quantification of AST and ALT**

Total AST and ALT levels in serum of iAST control mice and tumor-bearing mice (day 56 after virus injection) were measured on COBAS c system (c311, Roche/Hitachi). Therefore, commercially available ALTL (20764957 322) and ASTL (20764949 322) kits were used.
