# Supplementary figures and images for: Characterizing responsive and refractory orthotopic mouse models of hepatocellular carcinoma in cancer immunotherapy
Source: PLoS One. 2019 Jul 10;14(7):e0219517. doi: 10.1371/journal.pone.0219517 (PMC6619768; doi:10.1371/journal.pone.0219517)

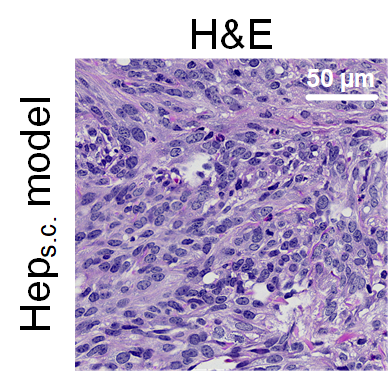

Supplement: S1 Fig — H&E staining of subcutaneous Hep-55.1c tumor of day 21 after cell inoculation. (TIF) [file pone.0219517.s002.tif]

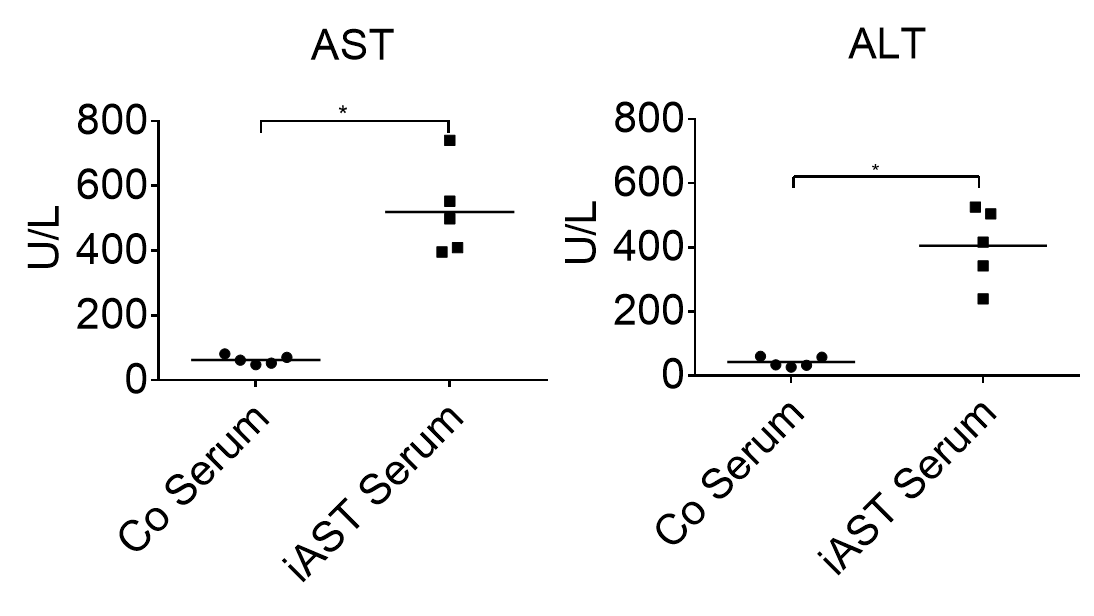

Supplement: S2 Fig — Analysis of liver enzymes AST and ALT in serum of iAST control mice and tumor-bearing mice of day 56 after virus injection. (TIF) [file pone.0219517.s003.tif]

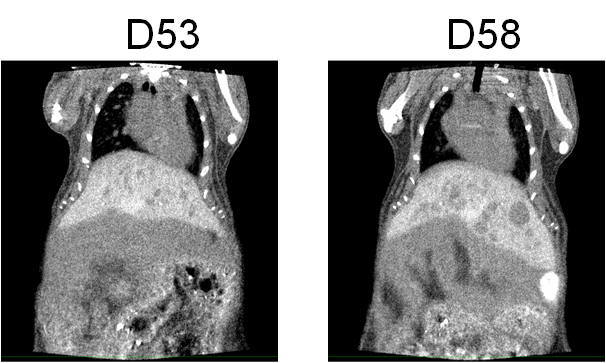

Supplement: S3 Fig — Representative μCT images of an iAST mouse on day 53 and day 58 after virus injection. (TIF) [file pone.0219517.s004.tif]

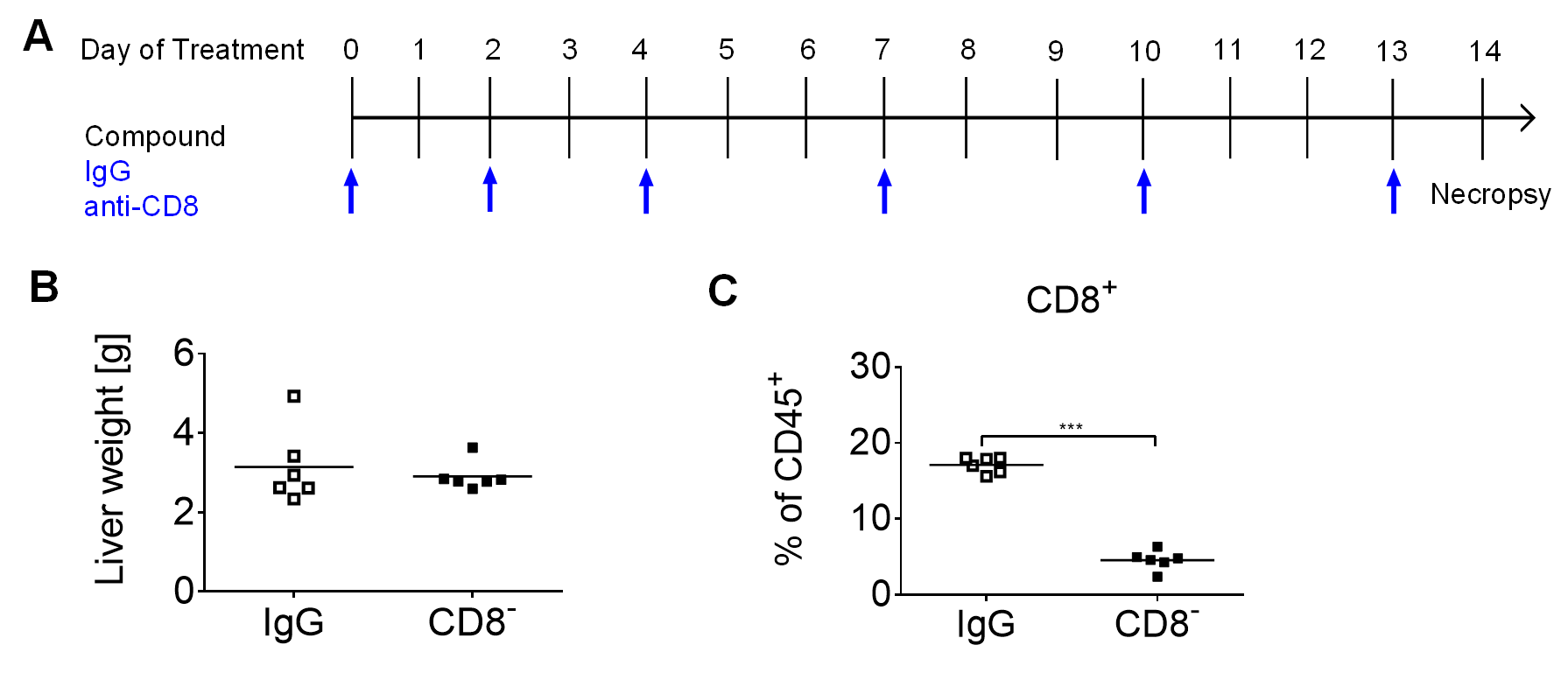

Supplement: S4 Fig — A, Treatment schedule illustrating the days of CD8+ T cell depletion (Day 0, 2, 4, 7, 10, 13) using a CD8α T cell depletion antibody (4 mg/kg, i.p.). Necropsy of mice was performed at day 14 after treatment initiation. B, Assessment of the weight of explanted livers including multinodular HCC tumors did not show a change in tumor load after depletion compared to control IgG (n = 6). C, Flow cytometry analysis confirms the successful depletion of CD8+ T cells in iAST tumors. B-C, Comparisons between groups were performed by Student’s t-test (***p<0.001). (TIF) [file pone.0219517.s005.tif]

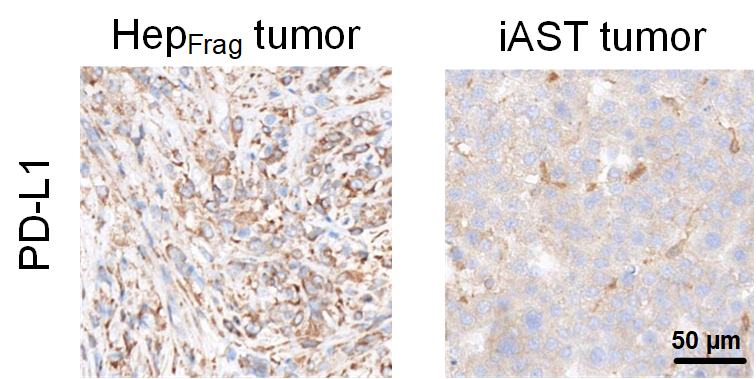

Supplement: S5 Fig — Representative tumor sections of HepFrag (day 28) and iAST tumors (day 56) were stained for PD-L1. A higher PD-L1 expression was observed in HepFrag tumors as compared to iAST tumors. (TIF) [file pone.0219517.s006.tif]
